# Allele-Specific Interactions between *CAST AWAY* and *NEVERSHED* Control Abscission in *Arabidopsis* Flowers

**DOI:** 10.3389/fpls.2016.01588

**Published:** 2016-10-21

**Authors:** William D. Groner, Megan E. Christy, Catherine M. Kreiner, Sarah J. Liljegren

**Affiliations:** Department of Biology, University of Mississippi, OxfordMS, USA

**Keywords:** abscission, cell separation, shedding, NEV, CST, ARF GAP, RLCK

## Abstract

An advantage of analyzing abscission in genetically tractable model plants is the ability to make use of classic genetic tools such as suppression analysis. We have investigated the regulation of organ abscission by carrying out suppression analysis in *Arabidopsis* flowers. Plants carrying mutations in the *NEVERSHED* (*NEV*) gene, which encodes an ADP-ribosylation factor GTPase-activating protein, retain their outer floral organs after fertilization. Mutant alleles of *CAST AWAY* (*CST*), which encodes a receptor-like cytoplasmic kinase, were found to restore organ abscission in *nev* flowers in an allele-specific manner. To further explore the basis of the interactions between *CST* and *NEV*, we tested whether the site of a *nev* mutation is predictive of its ability to be suppressed. Our results suggest instead that the strength of a *nev* allele influences whether organ abscission can be rescued by a specific allele of *CST*.

## Introduction

Plants have the astonishing ability to release their floral organs, leaves, fruit, and seeds at programmed points in their life cycle or in response to signals from their environment. Within *Arabidopsis* flowers, the series of events leading to organ abscission is genetically tractable. Analysis using this model system has revealed the influence of organ boundary genes in establishing the placement of abscission zones (Wang et al., 2006; González-Carranza et al., 2007; Gómez-Mena and Sablowski, 2008; McKim et al., 2008; Gubert et al., 2014), the critical roles played by hormones such as jasmonic acid (Kim et al., 2013) and managers of membrane traffic (Liljegren et al., 2009; Liu et al., 2013), and a signaling module that regulates the cell separation phase of organ abscission (Fang and Fernandez, 2002; Cho et al., 2008; Stenvik et al., 2008; Shi et al., 2011; Gubert and Liljegren, 2014; Patharkar and Walker, 2015; Santiago et al., 2016; Taylor et al., 2016). Central components in this module include a secreted peptide, INFLORESCENCE DEFICIENT IN ABSCISSION (IDA) and redundant leucine-rich repeat receptor-like kinases, HAESA (HAE) and HAESA-like2 (HSL2), that activate a MAP kinase cascade leading to organ abscission.

**FIGURE 1:**
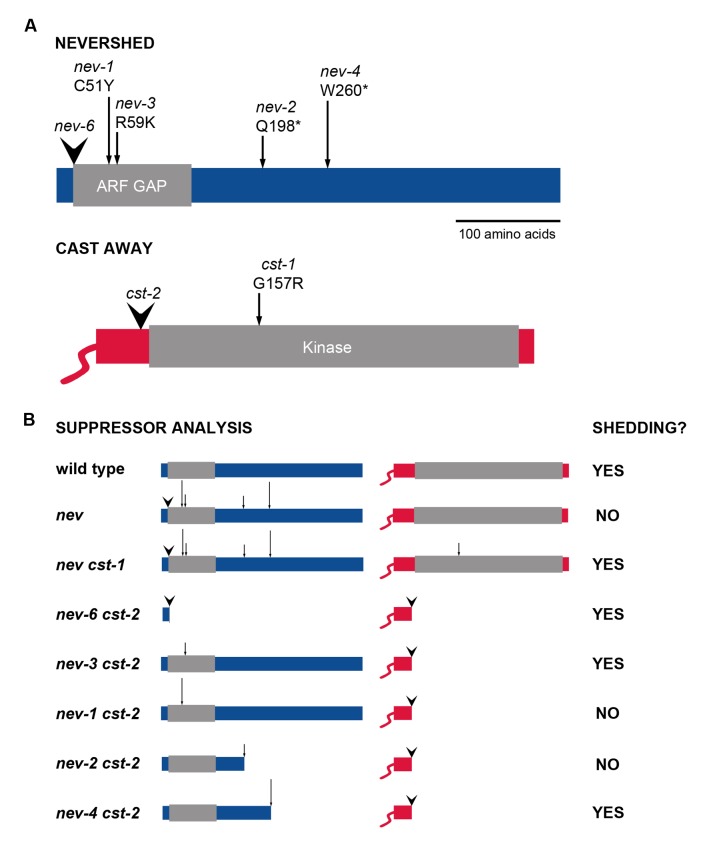
**Alleles of *NEV* and *CST* used in suppressor analysis of organ abscission.** **(A)** The sites of the mutations analyzed are indicated within the encoded NEV and CST proteins (Liljegren et al., 2009; Burr et al., 2011). T-DNA insertions are marked by arrowheads and point mutations by arrows. **(B)** Diagram of the *nev cst* allele combinations tested for rescue of organ shedding (Burr et al., 2011; this study).

We have used suppression analysis as a genetic tool to identify additional genes that control the abscission process in *Arabidopsis* flowers. Starting with the *nevershed* (*nev*) mutant which blocks organ shedding due to defects in membrane traffic (Liljegren et al., 2009), we screened for second-site mutations that would restore organ abscission in the presence of the original mutation. The *nev-3* allele chosen for this screen (**Figure 1A**) changes an invariant arginine in the encoded protein known to be essential for ADP-ribosylation factor GTPase-activating activity (Luo et al., 2007). Multiple alleles of genes encoding three receptor-like kinases—EVERSHED (EVR), SOMATIC EMBRYOGENESIS RECEPTOR-LIKE KINASE1 (SERK1), and CAST AWAY (CST)—were found to rescue abscission in *nev* flowers (Leslie et al., 2010; Lewis et al., 2010; Burr et al., 2011). Mutations in these receptor-like kinases are also able to reverse *nev*-mediated alterations in the structure of the Golgi apparatus and associated *trans*-Golgi network. Additional analyses suggest that activation of organ abscission is modulated by inhibitory interactions between CST and EVR with HAE/HSL2 (Burr et al., 2011; Gubert and Liljegren, 2014). Recent studies have demonstrated that SERK1 and two related receptor-like kinases act as co-receptors of HAE/HSL2 (Meng et al., 2016; Santiago et al., 2016). We have proposed that CST and EVR may prevent the signaling that leads to organ abscission by sequestering HAE/HSL2 at the cell surface and promoting their internalization prior to activation by IDA (Burr et al., 2011). As NEV is thought to function in the cycling of HAE/HSL2 to the plasma membrane, disruption of CST or EVR activity may restore organ abscission in *nev* flowers by shifting the balance of stabilized HAE/HSL2 receptors at the cell surface from an excessive pool of internalized, inactive receptors in endosomal compartments (Burr et al., 2011; Bryan et al., 2012; Liljegren, 2012).

Contrasting behaviors are shown by the pair of *cst* mutant alleles we identified with regard to their ability to rescue abscission in *nev* flowers (Burr et al., 2011). The *cst-1* allele introduces a missense mutation (G157R) near the ATP-binding site within the CST kinase domain (**Figure 1A**), abolishing the kinase activity of the mutant protein. Organ shedding in *nev-3. nev-2*, and *nev-6* flowers is recessively rescued by two copies of the *cst-1* allele (**Figure 1B**; Burr et al., 2011). The *cst-2* allele contains a T-DNA insertion immediately upstream of the kinase domain, and is predicted to encode a truncated protein (**Figure 1A**). One copy of *cst-2* dominantly restores organ abscission in *nev-3* and *nev-6* flowers, but *nev-2* flowers retain their organs even if both copies of *cst-2* are present (**Figure 1B**; Burr et al., 2011).

As these results were partially consistent with the allele-specific mechanism of conformational suppression, in which a suppressor mutation restores a physical interaction between two proteins, we designed a study to determine whether the location of a *nev* mutation would be predictive of its ability to be rescued by the *cst* alleles. Specifically, we tested whether alleles that independently affect either the ARF GAP domain or the C-terminal region of NEV would mimic the distinct interactions of *nev-3* and *nev-2* with *cst-2*.

## Materials and Methods

### Plants

The mutant alleles used in this study and methods for genotyping *cst-1* and *cst-2* have been described previously (Liljegren et al., 2009; Burr et al., 2011). *nev-1* and *nev-4* were genotyped as described in **Supplementary Table S1**. The *nev-1. nev-4*, and *cst-1* mutants were isolated from the L*er* ecotype; the *cst-2* mutant was isolated from the Col ecotype. Since the *nev cst-2* double mutants would be analyzed in a mixed L*er*/Col background, a *cst-1* stock backcrossed once into the Col ecotype was used to generate the *nev cst-1* double mutants. Plants were grown at 21°C with 50% humidity and a 16-h photoperiod.

### Imaging

Digital images were taken with a PowerShot SX160 IS (Canon, Melville, NY, USA) or Alpha Innotech gel documentation system (ProteinSimple, San Jose, CA, USA). Image brightness and contrast were adjusted with Photoshop CS6 (Adobe, Mountain View, CA, USA).

### RT/PCR

Wild-type and mutant inflorescences with flowers through stage 15 (Smyth et al., 1990) were ground in liquid nitrogen, and RNA was extracted using the RNeasy Plant Mini Kit (Qiagen, Venlo, Netherlands) according to the manufacturer’s instructions. Specific regions of wild-type and mutant cDNAs were synthesized using gene-specific primers (described in **Supplementary Table S2**) and SuperScript III reverse transcriptase (Thermo Fisher Scientific, Waltham, MA, USA) according to the manufacturer’s instructions. A subset of the RNA samples were pre-treated with DNase using the Ambion DNA-free Kit (Thermo Fisher Scientific, Waltham, MA, USA) prior to cDNA synthesis. To confirm the presence of the *cst-2* transcript, a second round of amplification was performed with a nested primer. In addition, replicates were carried out with and without reverse transcriptase.

## Results

### Allele-Specific Suppression of *nev*-Mediated Abscission Defects

Previously, we discovered that while one copy of the *cst-2* allele is sufficient to rescue organ shedding in *nev-3* (R59K) flowers, abscission in *nev-2* (Q198*) flowers cannot be restored by either one or two copies of *cst-2* (**Figure 1B**; Burr et al., 2011). Whereas the *nev-3* mutation affects an arginine residue essential for the enzymatic activity of the ARF GAP domain, the protein encoded by *nev-2* is predicted to be truncated downstream of the ARF GAP domain (**Figure 1A**; Luo et al., 2007; Liljegren et al., 2009). Both copies of the *cst-1* allele are required to suppress the abscission defects of *nev-3* and *nev-2* flowers (**Figure 1B**; Burr et al., 2011). Based on these results, we hypothesized that if CST and NEV function in a complex, the ARF GAP domain of NEV might facilitate this interaction (Burr et al., 2011).

**FIGURE 2:**
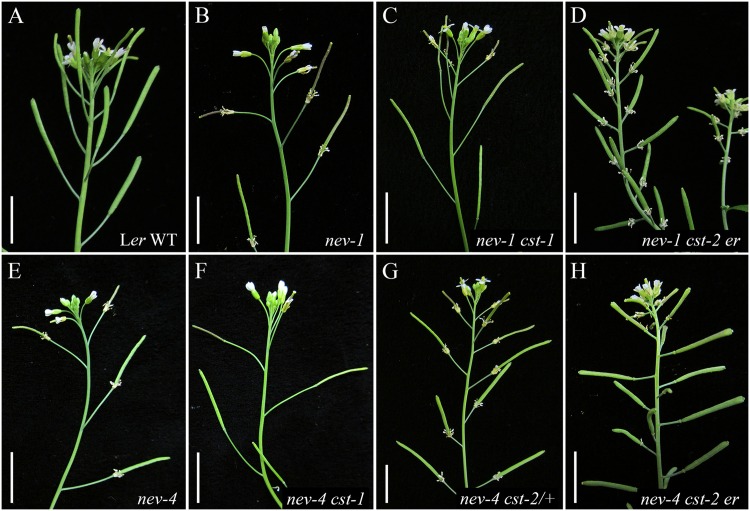
**Rescue of organ shedding in *nev cst* flowers is allele-dependent.** The outer organs of wild-type (WT) flowers are shed by floral stage 17 **(A)**, and stay attached in *nev-1* **(B)** and *nev-4* **(E)** flowers. Abscission is restored recessively in *nev-1 cst-1* **(C)** and *nev-4 cst-1* **(F)** flowers. Although it behaves dominantly in *nev-3* and *nev-6* flowers (Burr et al., 2011), the *cst-2* allele is unable to suppress the abscission defects of *nev-1* flowers **(D)**, and rescues organ shedding recessively in *nev-4* flowers **(G,H)**. Scale bars = 1 cm.

To investigate whether other *nev* alleles that alter critical residues in the ARF GAP domain show similar interactions with the *cst* alleles, we analyzed *nev-1 cst-1* and *nev-1 cst-2* double mutants (**Figure 2**). *nev-1* is a missense allele (C51Y) that alters the third essential cysteine within the Cys-x2-Cys-x(16,17)-Cys-x2-Cys zinc finger motif of the ARF GAP domain (**Figure 1A**; Liljegren et al., 2009). While *cst-1* is able to recessively suppress the shedding defects of *nev-1* flowers, the floral organs of the *nev-1 cst-2* double mutant remain firmly attached (**Figures 2A–D**). These results indicate that despite their close proximity within the ARF GAP domain and indistinguishable single mutant phenotypes, the *nev-1* and *nev-3* alleles do not behave equivalently when interacting with *cst-2* (**Figures 1A,B**).

We also tested whether another *nev* allele that introduces a stop codon downstream of the ARF-GAP domain exhibits similar interactions with the *cst* alleles. Like *nev-2. nev-4* is a nonsense allele (W260*) predicted to encode an abbreviated protein with an intact ARF-GAP domain (**Figure 1A**; Liljegren et al., 2009). As with all *nev* alleles tested, *cst-1* recessively rescues organ abscission in *nev-4* flowers (**Figures 2E,F**). However, unlike *nev-2 cst-2* flowers (**Figure 1B**), the shedding defects of *nev-4* flowers can also be suppressed with two copies of *cst-2* (**Figures 2G,H**). Therefore, despite the shared features of the *nev-2* and *nev-4* alleles, they interact with *cst-2* in distinct modes (**Figures 1A,B**).

### Analysis of *nev* and *cst* Transcripts

To examine whether there are qualitative differences in expression of the *nev* and *cst* mutant transcripts compared to wild-type, RT/PCR experiments were carried out on total RNA isolated from the inflorescences of wild-type and mutant plants. Oligos located in exon 11 of *NEV* and exon 6 of *CST* were used to synthesize the first strand of the cDNAs, and specific regions of the transcripts were subsequently amplified (see **Supplementary Table S2**). Substantial differences were not observed for the transcript levels of either missense (*nev-3. nev-1. cst-1*) or nonsense (*nev-4. nev-2*) alleles of *NEV* and *CST* compared to wild-type (**Supplementary Figures S1A** and **S2A**). In contrast, comparable levels of correctly spliced transcripts were not apparent in either of the insertional alleles (*nev-6. cst-2*) analyzed (**Supplementary Figures S1A** and **S2A**).

To test for the presence of altered transcripts in *nev-6* and *cst-2* flowers, oligos located upstream of the T-DNA insertion sites were used to synthesize cDNA fragments from DNase-treated RNA samples (**Supplementary Table S2**; **Supplementary Figures S1B** and **S2B**). Similar levels of a *NEV* cDNA product including part of exon 1 were observed in all *nev* mutants and wild-type plants examined (**Supplementary Figure S1B**). Since the *nev-6* T-DNA insertion is located in intron 1, these results indicate that an altered mutant transcript is produced in *nev-6* flowers that may encode an abbreviated protein. Reduced levels of nested products including parts of exon 1 and 2 of *CST* were observed in *cst-2* flowers compared to *cst-1* and wild-type (**Supplementary Table S2**; **Supplementary Figure S2B**). These results are consistent with the production of a truncated cst-2 protein.

## Discussion

Here we report further evidence that the *cst-1* and *cst-2* alleles differ in their ability to restore organ shedding in *nev* flowers. While *cst-1* recessively suppresses each of the five *nev* alleles tested, *cst-2* suppresses *nev-3* and *nev-6* dominantly, *nev-4* recessively, and fails to rescue *nev-1* and *nev-2* (Burr et al., 2011; this study).

These results highlight the complexity of interpreting the mechanisms of allelic suppression. Although the ultimate goal for many geneticists carrying out suppression analysis is to find instances of conformational suppression, whereby allele-specific rescue reflects a restored physical interaction between two mutant proteins, this scenario is actually rare in practice (Manson, 2000). Indeed, our selection of the *nev-3* missense allele (**Figure 1A**) as the genetic background for this screen was driven by an interest in identifying a mutant version of an unknown protein that might interact with and restore the ARF GAP enzymatic activity of the nev mutant protein.

Instead, our results suggest that the *cst-1* and *cst-2* alleles restore organ abscission in *nev* flowers through distinct suppression mechanisms. We have found that the kinase-dead CST protein encoded by *cst-1* recessively suppresses the abscission defects of all *nev* alleles tested, including *nev-6*, which is predicted to produce an abbreviated protein missing the ARF GAP domain (**Figure 1A**; **Supplementary Figure S1B**). Suppression of a deletion (or null) allele of the original gene by an extragenic suppressor is considered strong evidence of bypass suppression (Prelich, 1999). Bypass suppression occurs when a second site mutation creates an alternate opportunity to cover the function disabled by the first mutation (Manson, 2000; Michels, 2002). Another hallmark of bypass suppression is that it is not allele-specific (Manson, 2000), which fits with the observed behavior of the *nev cst-1* double mutants (**Figure 1B**). Considering that interactions between CST and HAE were detected in subdomains of the plasma membrane via biomolecular fluorescence complementation assays (Burr et al., 2011), the *cst-1* allele may consistently rescue organ shedding in *nev* flowers due to the failure of the kinase-deficient cst-1 protein to promote the internalization of the HAE/HSL2 receptors from the cell surface. The recessive nature of the *cst-1* suppression suggests that the reduced amount of the functional CST kinase in *nev* flowers heterozygous for *cst-1* is sufficient to remove enough of the HAE/HSL2 receptors from this plasma membrane pool to prevent activation of the MAP kinase module leading to organ abscission. Redelivery of HAE/HSL2 to the cell surface after internalization is predicted to be disrupted in each of the *nev* mutant alleles tested (**Figure 1A**).

We have found that *cst-2*, which may produce reduced levels of an abbreviated, membrane-associated protein without a kinase domain (**Figure 1A**; **Supplementary Figure S2B**), rescues organ abscission in *nev-3. nev-4*, and *nev-6* flowers but not in *nev-1* or *nev-2* flowers (**Figure 1B**). This allele-specific outcome may result from a dominant-negative mutation enacting a gradient of suppression (Manson, 2000; Burr et al., 2011). Under this scenario, the predicted strength of the *nev* alleles tested would range from *nev-6* and *nev-3* (relatively weak; rescued by one copy of *cst-2*) to *nev-4* (intermediate, rescued by two copies of *cst-2*) to *nev-1* and *nev-2* (strong, not rescued by *cst-2*). Relative differences in the activities of nev mutant proteins may impact the ratio of HAE/HSL2 receptors trapped in the endosomal compartments and thereby influence the ease of *cst-2* mediated suppression (**Figure 1B**). While a truncated nev-6 mutant protein without an ARF GAP domain would not be expected to retain more function than the nev-2 mutant protein (**Figure 1**), intronic T-DNA insertions can be spliced out in a fraction of the transcripts produced, leading to synthesis of functional protein (Chehab et al., 2011; Rodriguez et al., 2014). Although we did not detect notable levels of correctly spliced *NEV* transcripts in *nev-6* flowers (**Supplementary Figure S1A**), it is likely that even a small amount of functional protein is sufficient to promote abscission. Indeed, it has been previously observed that the petals of *nev-7* flowers detach more readily than those of *nev-3* flowers (Liu et al., 2013). Like *nev-6*, the *nev-7* allele contains a T-DNA insertion in the first intron (Liljegren et al., 2009).

It is striking that the *nev-1* and *nev-2* alleles can be recessively rescued by *cst-1* but not by *cst-2*. These results, in addition to the dominant suppression of *nev-3* and *nev-6* by *cst-2*, suggest that the truncated cst-2 protein may exhibit an altered set of interactions with receptor-like kinase complexes than the cst-1 protein. Future analysis of the expression, localization, and ability of the cst-2 and cst-1 mutant proteins to form heteromeric complexes with EVR and HAE may reveal additional clues to the unique mechanisms underlying their restoration of the signaling leading to organ abscission in *nev* flowers.

With the growing accessibility of approaches to identify the transcriptomes of abscission zone cells in model as well as crop plants using laser capture microdissection (Cai and Lashbrook, 2006, 2008; Agustí et al., 2009) and RNA sequencing (Niederhuth et al., 2013; Kim et al., 2016; Sundaresan et al., 2016), the agronomic value of using model plants to study abscission is under debate (Patterson et al., 2015). The recent discovery that drought-triggered leaf abscission is dependent on the activities of IDA, HAE/HSL2, and NEV (Patharkar and Walker, 2016) significantly enhances the usefulness of *Arabidopsis* as a model system. Furthermore, until analysis of gene function is feasible in crop plants, parallel approaches to investigate the functions of abscission zone-enriched genes in model plants with reverse genetic approaches will be crucial.

Forward genetic screens, when carefully designed, are also expected to provide novel insights regarding the regulation of organ abscission. In addition to our discovery of a set of receptor-like kinases that modulate organ abscission via proposed interactions with HAE and HSL2, the homeodomain transcription factor BREVIPEDICELLUS (BP) was found to act downstream of the IDA-HAE/HSL2 signaling module through suppression analysis of *ida* flowers (Shi et al., 2011). While *bp* mutants display enlarged abscission zones (Wang et al., 2006), it is noteworthy that the *cst. evr*, and *serk1* mutants do not present phenotypes on their own, yet alleles of each are able to rescue organ shedding in the context of *nev* flowers (Leslie et al., 2010; Lewis et al., 2010; Burr et al., 2011). Suppression analysis of a weak *hae hsl2* mutant has revealed that mutations in either of two mannosyltransferases that normally mediate degradation of the mutant hsl2 protein in the ER may restore abscission by allowing this partially functioning receptor to escape to the cell surface (Baer et al., 2016). Understanding the threshold levels at which organs are released in sensitized mutants like *nev. ida*, and *hae hsl2* may inform the future design of nuanced solutions to control abscission in crop plants.

## Author Contributions

SL designed the experiments. All authors performed the experiments and contributed in preparing the figures.

## Conflict of Interest Statement

The authors declare that the research was conducted in the absence of any commercial or financial relationships that could be construed as a potential conflict of interest.
