# Supplementary material for: Allele-Specific Interactions between CAST AWAY and NEVERSHED Control Abscission in Arabidopsis Flowers
Source: Front Plant Sci. 2016 Oct 21;7:1588. doi: 10.3389/fpls.2016.01588 (PMC5073242; doi:10.3389/fpls.2016.01588)
Supplement: Supplementary file 1 [file Table_1.DOCX]

**Table S1. Genotyping methods used in this study.**

| Allele | Enzyme | PCR  product  (bp) | Digest products  (bp) | | Oligos |
| --- | --- | --- | --- | --- | --- |
| *nev-1* | SphI or  PaeI | 611 | WT  *nev-1* | 337, 274  611 | 5’-CAATTCTGATAATTTTGAAGTTTTCTTG-3’  5’-CGACAATGGGAGTACAAG-3’ |
| *nev-4* | BspHI or  PagI | 602 | WT  *nev-4* | 602  301, 301 | 5’-CATTATTTTACTCACTGTGTTTTTAGAG-3’  5’-GCCTTTCACATCTTTTGGTG-3’ |
