# Supplementary material for: Allele-Specific Interactions between CAST AWAY and NEVERSHED Control Abscission in Arabidopsis Flowers
Source: Front Plant Sci. 2016 Oct 21;7:1588. doi: 10.3389/fpls.2016.01588 (PMC5073242; doi:10.3389/fpls.2016.01588)
Supplement: Supplementary file 2 [file Table_2.DOCX]

**Table S2. RT/PCR oligos used in this study.**

| Gene Region | Oligos (5’ to 3’) | cDNA  product (bp) | Genomic DNA product (bp) |
| --- | --- | --- | --- |
| *NEV*  exons 1-3 | NEV1: ATTTTGACCTCGTCGTCTCCGTAG  NEV2: TTCGATATGTGTACCCCGAGACTC | 293 | 1197 |
| *NEV*  exons 4-6 | NEV3: CTGCCACTCTGGACACATGGCTC  NEV4: GGGAAGATTTATTCTCGTTGCAGC | 350 | 550 |
| *NEV*  exons 8-11 | NEV5: GAAAATTGTCACAGCCAAGCCTGC  NEV6: GGCAAATTCAGAAAAAGTCTCTCTC | 666 | 1113 |
| *NEV*  exon 1 | NEV1: ATTTTGACCTCGTCGTCTCCGTAG  NEV7: GCATTAAGCTCCTTAGAGACGTTG | 131 | 131 |
| *CST*  exons 1-2 | CST1: ATCCACTAGCTCTTGTCTGAATTG  CST2: GACGAGAACTCTGTTCCATTGCTG | 189 | 298 |
| *CST*  exons 1-3 | CST1: ATCCACTAGCTCTTGTCTGAATTG  CST3: AGATTTCGGTGCGAAAGCATTCCC | 540 | 1253 |
| *CST*  exons 1-5 | CST1: ATCCACTAGCTCTTGTCTGAATTG  CST4: CATATGTGCCCATGATCC | 863 | 1758 |
| *CST*  exon 6 | CST5: GTTGTGCGCCGTCAGTCCGGTC | --- | --- |
| *CST*  exons 1-2 | CST6: AGGCTCATTAGAAATTGATG  CST2: GACGAGAACTCTGTTCCATTGCTG | 124 | 233 |
