# Supplementary figures and images for: Allele-Specific Interactions between CAST AWAY and NEVERSHED Control Abscission in Arabidopsis Flowers
Source: Front Plant Sci. 2016 Oct 21;7:1588. doi: 10.3389/fpls.2016.01588 (PMC5073242; doi:10.3389/fpls.2016.01588)

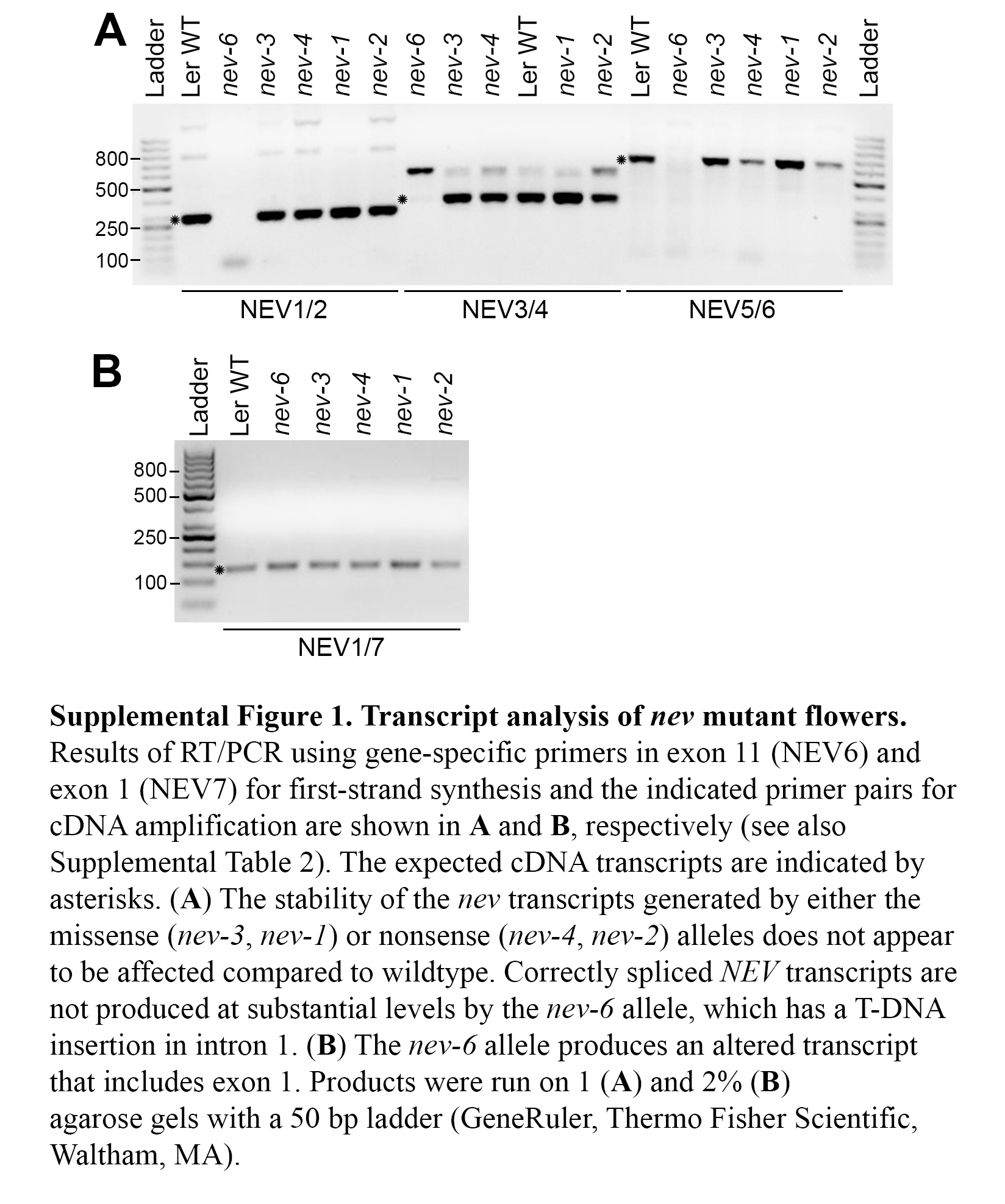

Supplement: Supplementary file 3 [file Image_1.TIF]

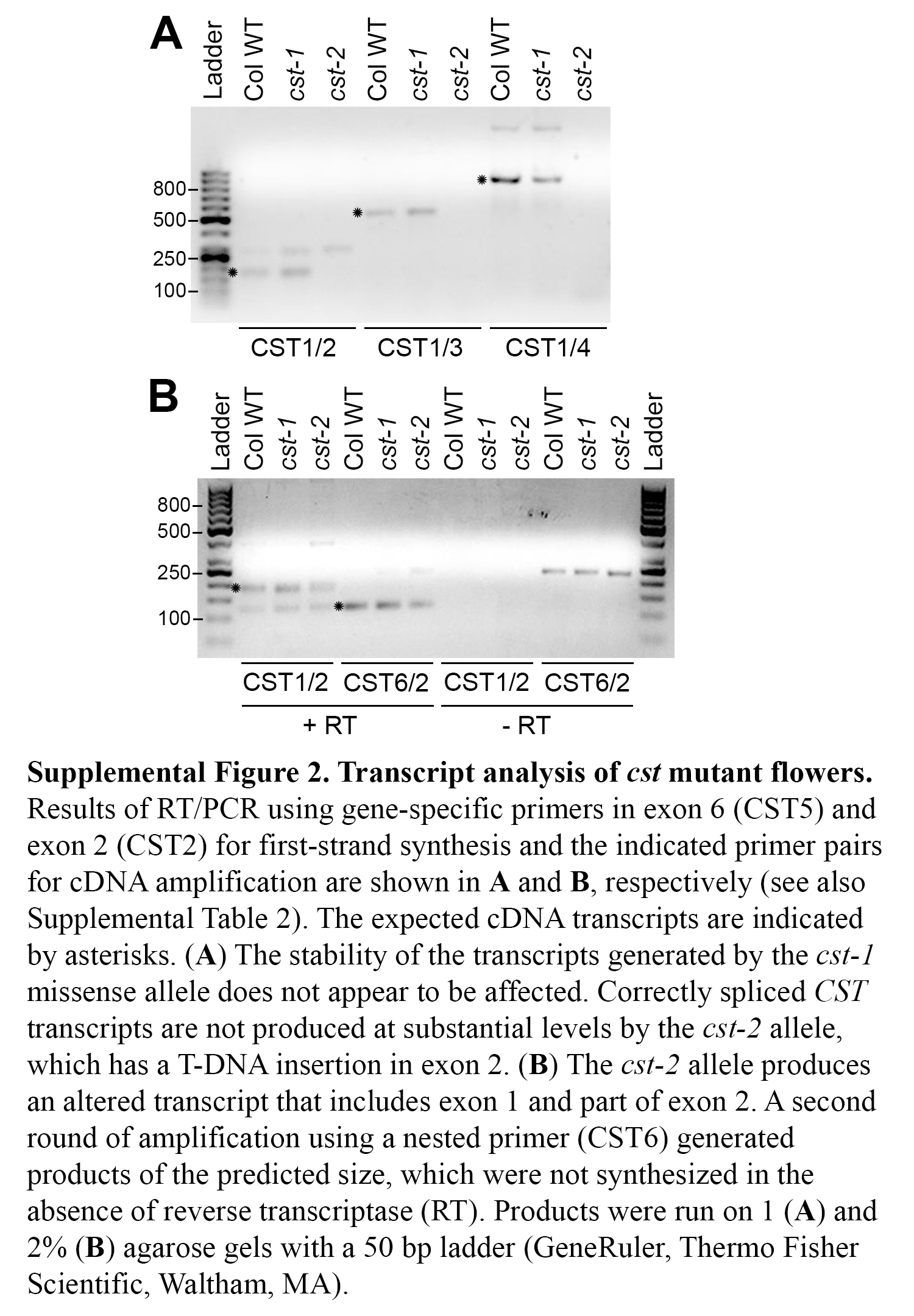

Supplement: Supplementary file 4 [file Image_2.TIF]
